# Estimating Risk of Chronic Pain and Disability Following Musculoskeletal Trauma in the United Kingdom

**DOI:** 10.1001/jamanetworkopen.2022.28870

**Published:** 2022-08-26

**Authors:** David W. Evans, Alison Rushton, Nicola Middlebrook, Jon Bishop, Marco Barbero, Jaimin Patel, Deborah Falla

**Affiliations:** 1 College of Life and Environmental Sciences, University of Birmingham, Edgbaston, Birmingham, United Kingdom; 2 School of Physical Therapy, University of Western Ontario, London, Ontario, Canada; 3 Department of Health Professions, Manchester Metropolitan University, Manchester, United Kingdom; 4 College of Medical and Dental Sciences, University of Birmingham, Edgbaston, Birmingham, United Kingdom; 5 Department of Business Economics, Health and Social Care, Rehabilitation Research Laboratory, University of Applied Sciences and Arts of Southern Switzerland, Lugano, Switzerland; 6 University Hospitals Birmingham, Birmingham, United Kingdom

## Abstract

**Importance:**

Serious traumatic injury is a leading cause of death and disability globally, with most survivors known to develop chronic pain.

**Objective:**

To describe early variables associated with poor long-term outcome for posttrauma pain and create a clinical screening tool for this purpose.

**Design, Setting, and Participants:**

This was a prospective cohort study at a major trauma center hospital in England. Recruitment commenced in December 2018 and ceased in March 2020. Participants were followed up for 12 months. Patients aged 16 years or older who were hospitalized because of acute musculoskeletal trauma within the preceding 14 days were included. Data were analyzed from March to December 2021.

**Exposure:**

Acute musculoskeletal trauma requiring admittance to a major trauma center hospital.

**Main Outcomes and Measures:**

A poor outcome was defined as Chronic Pain Grade II or higher and measured at both 6 months (primary time point) and 12 months. A broad range of candidate variables potentially associated with outcomes were used, including surrogates for pain mechanisms, quantitative sensory testing, and psychosocial factors. Univariable models were used to identify the variables most likely to be associated with poor outcome, which were entered into multivariable models. A clinical screening tool (nomogram) was derived from 6-month results.

**Results:**

In total, 1590 consecutive patients were assessed for eligibility, of whom 772 were deemed eligible and 124 (80 male [64.5%]; mean [SD] age, 48.9 [18.8] years) were recruited. At 6 months, 19 of 82 respondents (23.2%) reported a good outcome, whereas at 12 months 27 of 44 respondents (61.4%) reported a good outcome. At 6 months on univariable analysis, an increase in total posttraumatic stress symptoms (odds ratio [OR], 2.09; 95% CI, 1.33-3.28), pain intensity average (OR, 2.87; 95% CI, 1.37-6.00), number of fractures (OR, 2.79; 95% CI, 1.02-7.64), and pain extent (OR, 4.67; 95% CI, 1.57-13.87) were associated with worse outcomes. A multivariable model including those variables had a sensitivity of 0.93, a specificity of 0.54, and C-index of 0.92.

**Conclusions and Relevance:**

A poor long-term pain outcome from musculoskeletal traumatic injuries may be estimated by measures recorded within days of injury. These findings suggest that posttraumatic stress symptoms, pain spatial distribution, perceived average pain intensity, and number of fractures are good candidates for a sensitive multivariable model and derived clinical screening tool.

## Key Points

**Question:**

Can long-term poor pain outcome be estimated within days of traumatic injury?

**Findings:**

In this cohort study, which included a broad range of candidate variables potentially associated with pain outcomes in 124 patients hospitalized with traumatic injuries, a poor long-term pain outcome could be estimated within a multivariable model by measuring the number of fractures, average pain intensity, pain extent, and posttraumatic stress symptoms within days of injury. A clinical screening tool is presented.

**Meaning:**

These findings suggest poor long-term pain outcome following traumatic injuries can be estimated within days of injury, which could help clinicians improve pain management strategies.

## Teaser

This cohort study examines factors associated with poor long-term outcomes among patients who experienced traumatic injury.

## Introduction

Traumatic injury is a leading cause of death and disability globally.^1^ Most people with traumatic injuries severe enough to require hospital admittance are known to develop long-term sequalae.^2^ Pain is one such sequelae, with approximately two-thirds of these individuals developing chronic pain.^3,4,5,6,7,8,9,10,11^ Advances in the care of major trauma patients have improved survival rates.^12,13^ Consequently, an increasing incidence of posttrauma sequelae, including chronic disabling pain, seems inevitable.

Presently, mechanisms underlying the transition from acute to chronic posttrauma pain are not fully understood. Traumatic injuries differ from common nontraumatic musculoskeletal pain conditions, such as low back pain, in 3 important ways. First, traumatic injuries are always accompanied by a discernible causal event, whereas two-thirds of low back pain cases are not.^14^ Second, tissue damage is invariably present following trauma, and its extent and location are readily identifiable, whereas an unclear relationship with pain and tissue damage exists in low back pain.^15^ Third, posttraumatic stress symptoms are common and appear to play an important role in the development and maintenance of chronic posttrauma pain.^16,17,18,19,20,21^

Research^22,23,24,25,26,27^ across a range of musculoskeletal pain conditions has identified other variables associated with poor outcomes, including high pain intensity, spatial extent of pain, indicators of central sensitization, and the number of previous pain episodes, which are likely to play a role following traumatic injuries. Understanding which factors operate early after traumatic injury and how they might influence the development of chronic pain is, therefore, worthy of exploration. Such knowledge could facilitate the development and implementation of more effective early posttrauma interventions with the ultimate goal of preventing poor long-term outcomes. The aims of this study were to (1) describe early variables associated with poor long-term outcome for posttrauma pain and disability and (2) present a screening tool for estimating patients at risk of developing a poor long-term pain outcome.

## Methods

We conducted a prospective, observational, cohort study of patients admitted to a major trauma center hospital in Birmingham, England. Approval for the study was gained from the Wales 2 NHS Research Ethics Committee, and participants provided written consent. We adhered to the Strengthening the Reporting of Observational Studies in Epidemiology (STROBE) reporting guideline.^28^ Full methodological details have been described elsewhere.^29^

### Participants

**Figure 1.**
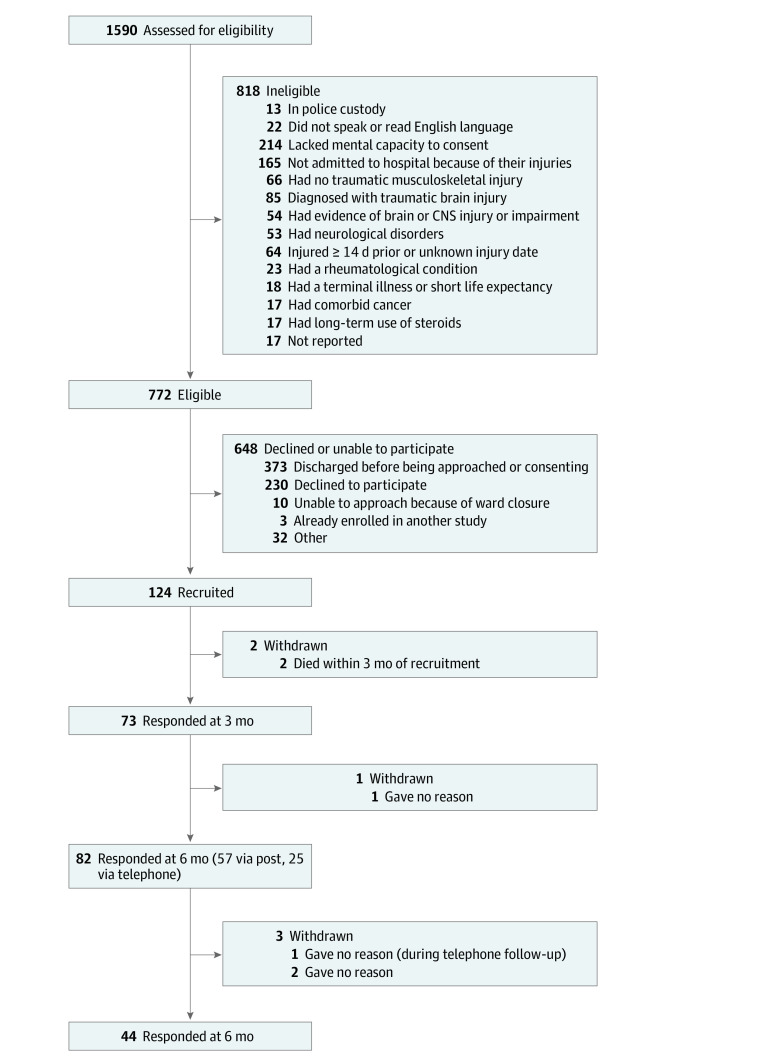
Participant Flow Through the Study CNS indicates central nervous system.

Consecutive potentially eligible patients were identified via the hospital admissions register between December 2018 and March 2020 (Figure 1). To be eligible, individuals had to be aged 16 years and older and admitted as inpatients to the major trauma center, with the main criteria for admission being acute musculoskeletal trauma within the preceding 14 days. Patients also had to have the capacity to use and understand written and verbal English language and the mental capacity to provide informed consent (eg, no confusion, delirium, severe cognitive impairment, or severe mental illness, defined by a score of ≤6 on the Abbreviated Mental Test).^30^ As much as possible, we wanted to limit inclusion to musculoskeletal traumatic injuries. Exclusions were therefore made if the patient had an acute intracranial lesion (eg, bleeding) combined with a score of 14 or less on the Glasgow Coma Scale, evident brain or central nervous system injury or impairment, long-term neurocognitive disorders (eg, brain tumor, multiple sclerosis, Alzheimer disease, or Parkinson disease), an ongoing rheumatological condition, prolonged use of corticosteroids, comorbid cancer, or terminal illness with short life expectancy.

After screening admission registers, research nurses visited potentially eligible patients within hospital wards. If a patient was interested in participating, the research nurse confirmed that they met the eligibility criteria, provided a copy of the study Participant Information Sheet, and answered any questions. In a minor amendment from our original protocol (because of reduced research nurse time availability), on the next available working day a university researcher would visit the patient in-ward to obtain their written consent and commence baseline data collection. Participants completed self-reported questionnaires at baseline, 3 months, 6 months (a priori primary time point), and 12 months. Additional physical assessments were performed at baseline only. Participants received usual care as required for their injuries (eg, pain medications, surgery, or rehabilitation).

### Definition of Outcome

Outcome was measured at 6 months (primary time point) and 12 months using the Chronic Pain Grade Scale (CPGS).^31^ At each time point, CPGS responses were classified into 1 of 5 ordinal chronic pain grades: no pain (grade 0), low disability and low-intensity pain (grade I), low disability and high-intensity pain (grade II), high disability and moderately limiting intensity pain (grade III), and high disability and severely limiting intensity pain (grade IV). Consistent with previous studies^32,33,34,35,36^ we defined a poor outcome on the CPGS as chronic pain grade II or higher.

### Candidate Variables

The range of candidate variables measured at baseline (see eTable 1 in the Supplement for full list) was intended to be as comprehensive as possible, encompassing psychosocial factors (eg, anxiety, depression, posttraumatic stress symptoms, and pain self-efficacy) and surrogates for the 4 primary mechanistic categories of pain: nociceptive (eg, injury severity and number of fractures), neuropathic (eg, painDETECT questionnaire), inflammatory markers (eg, C-reactive protein level), and nociplastic (eg, quantitative sensory testing and pain extent).

### Statistical Analysis

Data were analyzed with R statistical software version 4.0.2 (R Project for Statistical Computing). For all baseline characteristics and candidate variables, summary statistics (eg, means and SDs, medians and IQRs, or frequencies and percentages) are presented. Differences between participants achieving poor or good outcomes at 6 months and 12 months (eg, mean differences, odds ratios [ORs]) are presented along with 95% CIs and *P* values from 2-sided Wald tests and t-tests, with *P* < .05 considered significant. The data were assessed for normality via distributional plots and appropriate data transformations, or nonparametric tests used as necessary. All analyses were performed using complete case data with no imputation of missing values.

Univariable logistic regression was used to evaluate the potential of each single-domain candidate variable at the 6-month and 12-month time points. Candidate variables were selected for a multivariable logistic regression model of each time point, according to the relative magnitude of estimated variable effect sizes from the univariable analyses of 6-month data (primary time point) and clinical judgement. The number of candidate variables entered into the multivariable models was determined by the quantity of available outcome data at 6 months. The rms (Regression Modeling Strategies) package for R (version 6.2) was used to perform logistic regression, model validation and calibration.^37^ The rms package was also used to derive a clinical screening tool, in the form of a nomogram (a visual tool that allows individual estimates of outcome for chosen baseline values of the variables), using data from the 6-month multivariable model. Model discrimination and accuracy were estimated using version 6.0 of the caret package for R.^38^ Data were analyzed from March to December 2021.

## Results

### Participants

A total of 124 eligible participants (80 male [64.5%]; 104 [83.9%] White; mean [SD] age, 48.9 [18.8] years) were recruited to the study and provided baseline data. Follow-up response rates were 73 participants (58.9%) at 3 months, 82 participants (66.1%) at 6 months, and 44 participants (35.5%) at 12 months. There were no significant baseline differences between follow-up responders and nonresponders (eTable 2 in the Supplement). The study flowchart is presented in Figure 1.

### Participant Characteristics

Table 1 displays baseline characteristics of the 124 participants. Of note, 114 participants (91.9%) sustained at least 1 fracture, with 50 (40.3%) sustaining more than 1 fracture. Similarly, 109 (87.9%) had undergone at least 1 surgery since being admitted to hospital. At baseline, the mean (SD) number of days since sustaining their traumatic injuries was 6.2 (3.6) days, or 5.7 (3.1) days since being admitted to the hospital.

**Table 1.** Participant Characteristics at Baseline

Characteristic	Participants, No. (%)
Age, mean (SD), y	48.9 (18.8)
Body mass index, mean (SD)^a^	27.9 (6.3)
Sex at birth	
Male	80 (64.5)
Female	44 (35.5)
Ethnicity	
Asian or Asian British	10 (8.1)
Black or Black British	6 (4.8)
Chinese or Chinese British	0
White	104 (83.9)
Other ethnic group^b^	3 (2.4)
Preferred not to say	1 (0.8)
Education age	
Age ≤16 y	48 (39.0)
Age 17-19 y	40 (32.5)
Age ≥20 y	30 (24.4)
Still in full-time education	4 (3.3)
Preferred not to say	1 (0.8)
Working (at baseline)	
Yes	78 (62.9)
No	44 (35.5)
Preferred not to say	2 (1.6)
Smoker	
Yes	21 (16.9)
No	91 (73.4)
Preferred not to say	12 (9.7)
Time since trauma, mean (SD), d	6.2 (3.6)
Time since admission, mean (SD), d	5.7 (3.1)
Hospital stay, mean (SD), d	17.6 (14.4)
Lowest Glasgow Coma Scale score, mean (SD)	14.7 (1.2)
Intensive care since injury	
Yes	9 (7.3)
No	115 (92.7)
Ventilated since injury	
Yes	5 (4.0)
No	119 (96.0)
Underwent surgery	
Yes	109 (87.9)
No	15 (12.1)
Sustained fracture(s)	
Yes	114 (92.6)
No	10 (8.1)
No. of fracture(s)	
0	10 (8.1)
1	64 (51.6)
2	23 (18.6)
3	12 (9.7)
4	7 (5.7)
5	8 (6.5)
Location of injuries^c^	
Upper limb	25 (20.2)
Lower limb	112 (90.3)
Back or neck	40 (32.3)
Chest or abdomen	20 (16.1)
Head or face	9 (7.3)
Mechanism of injury^c^	
Fall	61 (49.2)
Vehicle	45 (36.3)
Sport or recreation	9 (7.3)
Work	6 (4.8)
Violence	5 (4.0)
Other or unknown	8 (6.5)
Circumstances of injury	
Civilian	112 (90.3)
Military	12 (9.7)
Medical history^c^	
Pulmonary	17 (13.7)
Cardiac	35 (28.2)
Diabetes	9 (7.3)
Vascular	27 (21.8)
Thyroid	8 (6.5)
Hypercholesterolemia	11 (8.9)
Neurological	2 (1.6)
Cancer	4 (3.2)
Bone	7 (5.7)
Psychiatric	17 (13.7)

^a^ Body mass index is calculated as weight in kilograms divided by height in meters squared.

^b^ Other ethnicity responses were African (1 participant), mixed race (1 participant), and not stated (1 participant).

^c^ Multiple responses were possible.

Chronic Pain Grades calculated for participants responding to 6-month and 12-month questionnaires are summarized in eFigure 1 in the Supplement. Notably, at the 6-month (primary) time point, only 19 (23.2%) of the 82 respondents reported a good outcome (ie, grade I or 0) while 63 (76.8%) reported a poor outcome (ie, grade II and above). At the 12-month mark, 17 (38.6%) reported a poor outcome. Despite only 44 responses being available from the 12-month questionnaire, the absolute number of good outcomes reported was higher than at 6 months (27 vs 19), resulting in a much higher proportion (61.4%) of good outcomes at 12 months.

Baseline scores of study participants by good and poor outcomes at 6 months and 12 months respectively are summarized in eTable 3 in the Supplement. For the data drawn from hospital records, participants reporting a poor outcome at either 6 months or 12 months spent substantially more days in the hospital on average compared with those reporting a good outcome. There was a mean difference of 8.23 days (95% CI, 0.91 to 15.55 days; *P* = .03) at 6 months and a mean difference of 11.76 days (95% CI, 2.30 to 21.22 days; *P* = .02) at 12 months. The mean number of fractures recorded at baseline was greater in those reporting a poor outcome at both 6 months (mean difference, 0.80 fractures; 95% CI, 0.14 to 1.45 fractures; *P* = .02) and 12 months (mean difference, 0.60 fracture; 95% CI, –0.03 to 1.22 fractures; *P* = .06).

There was a significant difference in every baseline general health and psychological measure between those reporting a poor outcome at 6 months compared with those who did not. Baseline pain self-efficacy was significantly greater in those reporting a good outcome at 12 months compared with those reporting a poor outcome (mean difference, 13.36 points; 95% CI, 3.67 to 23.05 points; *P* = .01). Likewise, all scores derived from the Impact of Events Scale–Revised (IES–R) were higher (ie, more severe symptoms) in those with a poor outcome at 12 months vs those with a good outcome. There were mean differences of 12.68 points (95% CI, 0.31 to 25.04 points; *P* = .04) for the total score, 0.56 points (95% CI, 0.00 to 1.12 points; *P* = .049) for the avoidance subscale, 0.66 points (95% CI, 0.01 to 1.31 points; *P* = .045) for the hyperarousal subscale, and 0.47 (95% CI, –0.15 to 1.08 points; *P* = .14) for the intrusion subscale.

With the exception of current pain intensity, baseline values of all pain-related measures were consistently greater in those reporting a poor outcome at both time points. These differences did not reach statistical significance at 12 months, however. There was also no significant difference in either 6-month or 12-month outcome groups for baseline values of sleep quality, C-reactive protein, or pain thresholds of any modality (pressure, heat, or cold) and location (local or remote to site of injury). However, baseline painDETECT questionnaire scores were significantly higher (ie, an increased likelihood of neuropathic pain) in those reporting a poor outcome at both 6 months (mean difference, 5.85 points; 95% CI, 1.35 to 10.35 points; *P* = .01) and 12 months (mean difference, 7.20 points; 95% CI, 2.10 to 12.30x points; *P* = .01).

### Univarible Logistic Regression Analyses

Results from the univariable logistic regression models of single-domain candidate variables of poor outcome at 6 months are displayed in Table 2. Candidate variables are ranked according to the magnitude of their estimated ORs. ORs are based on changes in scale (specified in the second column of Table 2), the magnitude of which was chosen so that proportional changes were approximately equivalent for each candidate variable. Univariable variables from the domains of posttraumatic stress (avoidance, hyperarousal, and intrusion) and pain spatial distribution (pain extent and pain region count) exhibited the largest ORs. Perceived average pain intensity and number of fractures were also univariable variables of poor outcome. The best fitting univariable model for average pain intensity modeled average pain intensity as a nonlinear term (a restricted cubic spline), meaning that the outcomes associated with an increase in average pain intensity varied depending on the initial value.

**Table 2.** Univariable Variables Associated With Poor Outcome Ranked by 6-Month Point Estimate

Candidate variable	Change in scale	Domain	OR (95% CI)	
			6 mo	12 mo
IES–R				
Avoidance subscale	1	Posttraumatic stress	5.23 (1.89-14.46)	1.96 (0.96-3.98)
Hyperarousal subscale	1	Posttraumatic stress	3.25 (1.28-7.09)	1.79 (0.98-3.26)
Pain intensity average	1 (2.5 to 3.5)	Perceived intensity of pain	2.87 (1.37-6.00)	1.05 (0.56-1.97)
No. of fractures	1	Tissue damage	2.79 (1.02-7.64)	1.79 (0.91-3.51)
Pain extent	5%	Pain spatial spread	4.67 (1.57-13.87)	1.52 (0.73-3.17)
IES–R				
Intrusion subscale	1	Posttraumatic stress	2.64 (1.33-5.23)	1.58 (0.85-2.94)
Total score	10	Posttraumatic stress	2.09 (1.33-3.28)	1.37 (0.99-1.89)
Pain intensity worst	1	Perceived intensity of pain	2.01 (1.28-3.16)	1.19 (0.86-1.65)
Penetrating injury	Yes	Tissue damage	2.01 (0.60-6.81)	0.61 (0.14-2.79)
painDETECT questionnaire	5	Neuropathic pain	1.90 (1.09-3.29)	2.48 (1.12-5.47)
Tampa Scale of Kinesiophobia-11	5	Fear of movement/activity	1.63 (1.09-2.45)	1.13 (0.76-1.67)
Pain region count	1	Pain spatial spread	1.50 (1.16-1.93)	1.07 (0.93-1.24)
Hospital Anxiety and Depression Scale				
Depression subscale	2	Depression	1.48 (1.09-2.02)	1.09 (0.83-1.43)
Anxiety subscale	2	Anxiety	1.39 (1.06-1.83)	1.04 (0.83-1.31)
Pain Self-Efficacy Questionnaire	–5	Pain self-efficacy	1.39 (1.14-1.69)	1.38 (1.05-1.81)
C-reactive protein	50 mg/L	Inflammation	1.28 (0.73-2.25)	0.89 (0.53-1.50)
Injury Severity Score	5	Tissue damage	1.39 (0.85-2.28)	1.12 (0.70-1.79)
Pain intensity now	1	Perceived intensity of pain	1.17 (0.91-1.50)	1.21 (0.90-1.63)
Sleep quality average	–1	Sleep	1.11 (0.90-1.37)	1.04 (0.82-1.32)
PPT (local)	250 kPa	Tissue sensitivity	1.10 (0.54-2.22)	1.31 (0.45-3.90)
CPT (local)	10 °C	Tissue sensitivity	1.10 (0.65-1.85)	1.53 (0.78-3.02)
Pain intensity least	1	Perceived intensity of pain	1.07 (0.82-1.41)	1.08 (0.81-1.45)
Body mass index	2	Body mass	1.03 (0.86-1.23)	1.39 (1.01-1.91)
Sleep last 24 h	–1	Sleep	1.02 (0.83-1.26)	1.07 (0.84-1.37)
HPT (remote)	4 °C	Tissue sensitivity	0.98 (0.52-1.85)	0.67 (0.28-1.63)
PPT (remote)	250 kPa	Tissue sensitivity	0.89 (0.50-1.59)	1.03 (0.49-2.19)
HPT (local)	4 °C	Tissue sensitivity	0.84 (0.42-1.69)	0.72 (0.27-1.91)
Pain intensity average	1 (6.6 to 7.5)	Perceived intensity of pain	0.73 (0.39-1.39)	1.47 (0.74-2.92)
CPT (remote)	10 °C	Tissue sensitivity	0.72 (0.43-1.22)	1.00 (0.50-2.00)

Abbreviations: CPT, cold pain threshold; HPT, heat pain threshold; IES–R, Impact of Event Scale–Revised; PPT, pressure pain threshold.

With few participants reporting a good outcome at 6 months, our selection of variables for the multivariable logistic regression models had to be parsimonious. Given that 2 of the 3 subscales of the IES–R had the largest univariable point estimates, we used the total IES–R score in the multivariable models. Additionally, because of a strong correlation between baseline values of pain extent and pain region count (Pearson correlation, *r* = 0.75; 95% CI, 0.63 to 0.83; *P* < .001), both of which represent the domain of pain spatial spread, we selected only pain extent for the multivariable models. Finally, given their relatively large univariable point estimates at 6 months, perceived average pain intensity and number of fractures were also included in the multivariable models. All 4 variables are continuous and were included in the models as linear additive terms with no interactions.

Table 3 displays the ORs of a poor outcome based on a unit change of each variable included in the multivariable logistic regression models. The multivariable model produced odds ratios of 3.18 (95% CI, 0.52-19.61) for number of fractures, 1.61 (95% CI, 0.96-2.70) for average pain intensity, 1.14 (95% CI, 0.92-1.41) for pain extent, and 1.04 (95% CI, 0.99-1.10) for posttraumatic stress symptoms. At 12 months, equivalent values were 1.65 (95% CI, 0.77-3.55) for number of fractures, 0.97 (95% CI, 0.67-1.40) for average pain intensity, 1.06 (95% CI, 0.92-1.23) for pain extent, and 1.03 (95% CI, 0.99-1.07) for posttraumatic stress symptoms. The final 6-month multivariable model was based on 67 participants providing data for the outcome and all 4 variables; 13 had a good outcome and 54 had a poor outcome. The final 12-month multivariable model was based on 39 participants providing data for the outcome and all 4 included variables.

**Table 3.** Effect Sizes From Multivariable Logistic Regression Models, Based on Variable Unit Changes

Variable	6 mo		12 mo	
	OR poor outcome (95% CI)	*P* value	OR poor outcome (95% CI)	*P* value
No. of fractures	3.18 (0.52-19.61)	.21	1.65 (0.77-3.55)	.19
Average pain intensity	1.61 (0.96-2.70)	.07	0.97 (0.67-1.40)	.86
Pain extent	1.14 (0.92-1.41)	.23	1.06 (0.92-1.23)	.41
Impact of Events Scale–Revised total	1.04 (0.99-1.10)	.11	1.03 (0.99-1.07)	.21

Abbreviation: OR, odds ratio.

The 6-month model achieved a sensitivity of 0.93, specificity of 0.54, positive predictive value of 0.89, negative predictive value of 0.64, area under the receiver operating characteristic curve (C-index) of 0.92, and a Brier score of 0.09 (eTable 4 in the Supplement). Following 999 bootstrap resamples, the optimism-corrected estimate of the C-index was calculated to be 0.89, suggesting model discrimination was still reasonably high after correcting for over-fitting. The optimism-corrected estimate of Nagelkerke *R*^2^ was 0.44. A calibration plot (eFigure 2 in the Supplement) revealed that the model was underestimating the low-to-middle probabilities of obtaining a poor outcome but overestimating the upper-middle range of probabilities between 0.75 and 0.90. This model was therefore good at identifying participants with a poor outcome at 6 months, but poor at identifying participants with a good outcome. By contrast, the 12-month model achieved a sensitivity of 0.53, specificity of 0.82, positive predictive value of 0.69, negative predictive value of 0.69, area under the receiver operating characteristic curve (C-index) of 0.78, and a Brier score of 0.20. Following 999 bootstrap resamples, the optimism-corrected estimate of the C-index was calculated to be 0.68, suggesting that model discrimination was still relatively low after correcting for overfitting. A calibration plot (eFigure 3 in the Supplement) revealed that the model was overestimating the low-to-middle probabilities of obtaining a poor outcome but underestimating the upper-middle range of probabilities between 0.25 and 0.65. This model was poor at identifying participants with a poor outcome at 12 months, but reasonably good at identifying participants with a good outcome.

### Clinical Screening Tool

**Figure 2.**
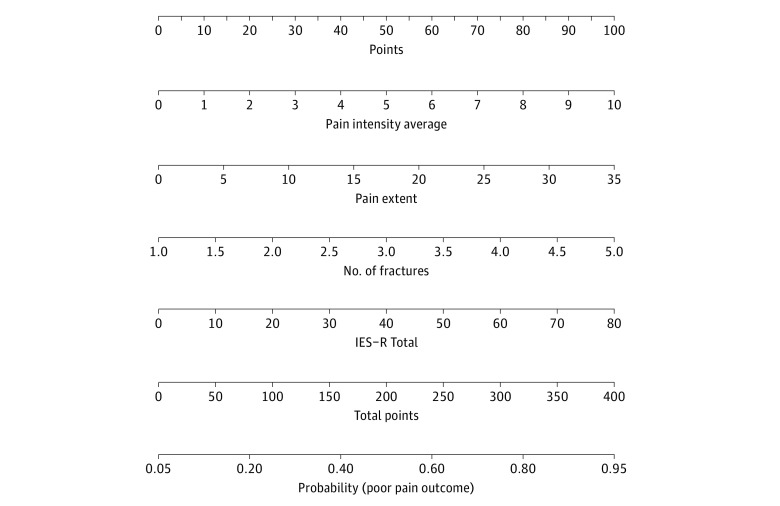
Nomogram for Calculating the Probability of Poor Outcome at 6 Months The interpretation of the nomogram is fairly simple. For example, an injured patient with an average pain intensity of 4 would score 40 points for that variable. If they also had 2 fractures, a pain extent of 5% and an Impact of Events Scale–Revised (IES–R) score of 22, they would score a further 24, 14, and 20 points respectively, producing a total score of 98 points. A straight vertical line from 98 total points down to the fitted probabilities at the bottom of the nomogram corresponds to a probability of a poor outcome at 6 months of around 83%.

A clinical screening tool (nomogram) was derived from the 6-month multivariable model (Figure 2). To use this screening tool, points can be calculated for each variable by selecting the appropriate baseline value and allocating points at the top of the plot. Once points for each variable have been summed to produce a total score, a straight vertical line from the total points to the fitted probabilities will correspond to the probability of a poor outcome at 6 months. Points are presented in eTable 5 in the Supplement.

## Discussion

The primary aim of this cohort study was to estimate a poor outcome for chronic pain and disability following musculoskeletal trauma. A large majority (76.8%) of respondents reported a poor outcome (chronic pain grade II or above) at 6 months, twice as high as the proportion of chronic pain in the general population.^39^ The equivalent figure at 12 months was significantly lower (38.6%), yet this is still a large proportion of respondents to be reporting chronic disabling pain 1 year after being injured. Recovery of posttrauma pain can therefore be summarized as very slow and by no means certain for all, a message consistent with previous studies.^5,6,7,40^

### Estimating Poor Outcome

Our results confirm that a poor long-term outcome from musculoskeletal traumatic injuries can be estimated by measures recorded within days of injury. Compared with other studies looking to estimate posttrauma pain outcome,^5,6,7,10,11,41^ we used a broad range of candidate variables. This approach appears to have been justified, since we found that the domains most likely to be associated with a poor outcome, on the basis of univariable point estimates at 6 months were posttraumatic stress symptoms, pain spatial distribution, pain intensity, and number of fractures. Measures of these domains were therefore selected for the multivariable models and subsequent screening tool.

With the exception of number of fractures, the domains most likely to be associated with a poor outcome are potentially modifiable. Even so, the presence of fractures following traumatic injury appears to be important. More than 90% of our sample had sustained 1 or more fracture, but the mean number of fractures was higher in those with poor outcome at both 6 months and 12 months. There was a mean difference of 0.80 fractures (95% CI, 0.14 to 1.45; *P* = .02) between those reporting a poor or good outcome at 6 months, and a mean difference of 0.60 fractures (95% CI, –0.03 to 1.22; *P* = .06) between those reporting a poor or good outcome at 12 months. Previous studies have also shown that chronic pain commonly develops following fractures^42,43,44,45,46,47^ and that their incidence greatly increases the chances of developing chronic widespread pain,^48^ neuropathic pain,^49,50^ and complex regional pain syndromes,^51^ all of which resist routine pain treatments. Although not modifiable, increased attention for immediate posttrauma pain management might be important for patients with multiple fractures.

Of the psychological constructs that we measured, posttraumatic stress symptoms were by far the univariable variables most likely to be associated with poor long-term outcomes. Indeed, 2 of the 3 IES–R subscales (avoidance and hyperarousal) were more likely than any other univariable variable to be associated with poor long-term outcomes (Table 3). This finding echoes previous studies in trauma-injured populations.^16,17,18,19,20,21^ Hence, attention ought to be given to these symptoms as an indicator for early posttrauma pain management. Indeed, although posttraumatic stress symptoms are expected to resolve for most,^11^ they may still have importance for estimating musculoskeletal pain long after traumatic injuries have been sustained.^52,53^

The relative value of pain spatial spread and pain intensity should not be a surprise. Pain spatial spread has been associated with long-term outcome in several previous studies, both in pain following physical trauma^54,55^ and in other musculoskeletal conditions.^56,57,58,59^ Both pain extent (the percentage of area shaded within a standardized body chart) and the number of painful regions (a count of predefined regions within a standardized body chart) were shown to have univariable value for estimating in this study. Although each is likely to measure a different aspect of the spatial distribution of pain, we chose just 1 (pain extent) for our multivariable models because their baseline values were correlated. Likewise, pain intensity measured (via a numerical rating scale) soon after injury has previously been shown to have long-term value in estimating outcomes.^26,60,61,62^ Of the variants of pain intensity (perceived current, average, worst, and least), perceived average was the univariable factor most likely to be associated with long-term poor outcomes. Interestingly, the best fitting model fitted perceived average pain intensity as a nonlinear term (modeled as a restricted cubic spline), implying that the outcomes associated with an increase in average pain intensity vary depending on its initial value. Others have noted nonlinearity of pain intensity measurements.^63^ Nevertheless, given its simplicity, interpretability, and widespread use, it remains a sensible choice for the multivariable models and subsequent screening tool.

With fewer participants than anticipated, we had to make pragmatic decisions regarding the retention of variables for multivariable models. The addition of some variables might have increased the accuracy our multivariable models. For example, signs and symptoms indicating a neuropathic pain component (ie, painDETECT) appear to have useful value for estimating outcomes in a posttrauma population as these were consistently associated with outcomes at both 6 months and 12 months. As with other musculoskeletal pain conditions,^64^ pain self-efficacy also showed utility in estimating outcomes. These measures should certainly be considered in future studies.

We expected other variables to have utility in estimating outcomes, but they did not. A strong inflammatory response is known to be triggered in the immediate aftermath of a traumatic injury. It was therefore surprising that C-reactive protein values did not appear to offer any value in estimating outcomes in this sample. This may be because we used only a single value, recorded closest to the time of the baseline questionnaire, whereas values of C-reactive protein are known to vary within the first week following traumatic injuries^65^; perhaps a characteristic of this change would be more useful than absolute values. Also surprising given the results of other studies,^66,67,68^ neither local nor remote pain thresholds, measured using any stimulus modality (heat, cold, or pressure), offered value in estimating outcomes. This could be because accurate topical measurements of tissue sensitivity depend on access to the skin surface at specific sites, which is often impeded by casts or dressings in the immediate aftermath of a traumatic injury. Equally unexpected given previous literature,^69,70,71^ perceived quality of sleep was not associated with outcomes, perhaps because this was recorded within days of the injuries (ie, before sleep issues could have an effect) and because we used a subjective measure of sleep quality.^72^

### Screening Tool

The second aim of this study was to create a clinical screening tool for estimating which patients were at risk of developing long-term posttraumatic pain and disability at 6 months. The results from this study have enabled the creation of such a screening tool (a nomogram) that can accurately estimate a poor outcome in individuals recently sustaining traumatic musculoskeletal injuries. The challenge in future studies will be to optimize this screening tool so that it can also accurately estimate individuals likely to have a good outcome (ie, improved specificity).

### Limitations

This study has limitations. Consistent with previous studies,^32,33,34,35^ we dichotomized the pain experience of participants measured using the CPGS into good or poor categories. Information and statistical power are lost when dichotomizing outcomes.^73^ However, given that the CPGS is designed to combine multidimensional pain items into meaningful categories, beyond pain duration,^31^ we believe that our definition of pain recovery was justifiable for this study.

The accuracy of our statistical models and accompanying screening tool was limited by the available sample size and will need to be confirmed in a future, larger data set. Recruiting patients soon after they have sustained traumatic injuries is clearly challenging. Participant numbers were lower than expected, due primarily to (1) more patients meeting our exclusion criteria than expected and (2) a premature cessation in hospital recruitment due to the emergence of the COVID-19 pandemic. We chose to exclude patients with substantial head injuries who were likely to have brain or central nervous system injury or impairment, or a formal diagnosis of traumatic brain injury. This was because traumatic brain injury has previously been associated with worse long-term outcomes in multiple domains^74^ and could have therefore dominated our models. Although we originally planned to recruit patients lacking mental capacity when they were first approached,^29^ we ended up not being able to do so (because of reduced research nurse capacity), resulting in a substantial proportion of potential participants being excluded (Figure 1). Ideally, these recruitment issues can be overcome in future studies, perhaps in part by including these factors as covariates rather than as exclusion criteria.

Participants with a poor outcome at both 6 months (mean difference of 8.23 days, 95% CI, 0.91 to 15.55; *P* = .03) and 12 months (11.76 days, 95% CI, 2.30 to 21.22; *P* = .02) were found to have spent more days in the hospital than those with a good outcome. Participants were recruited at a mean (SD) of 6.2 (3.6) days since their injuries, or 5.7 (3.1) days since being admitted to hospital. These figures are well within our eligibility criteria. Yet, it is possible that eligible but less severely injured patients were not recruited because they were discharged from the hospital before being approached by one of the research nurses (Figure 1), which could have influenced our results. Hospitalized trauma patients with earlier discharges were not recruited in previous studies.^10^ Hence, to avoid potential selection bias in future studies, we recommend that patients are recruited within the first 48 hours following their injuries.

## Conclusions

A poor long-term outcome from musculoskeletal traumatic injuries can be estimated by measures recorded within days of injury. Our results suggest that posttraumatic stress symptoms, pain spatial distribution, perceived average pain intensity, and number of fractures are good candidates for a sensitive multivariable model and derived clinical screening tool. Future work, with a larger number of participants, is required to improve the accuracy of statistical models and increase the specificity of screening tools to also estimate good outcomes.

## Notes

Supplement. **eFigure 1.** Chronic Pain Grade Scores at 6 Months and 12 Months Posttrauma **eFigure 2.** Calibration Plot for 6-Month Multivariate Model **eFigure 3.** Calibration Plot for 12-Month Multivariate Model **eTable 1.** Summary of Data Collected Within the Major Trauma Center **eTable 2.** Participant Baseline Characteristics by Participant Retention **eTable 3.** Participant Baseline Characteristics by Outcome **eTable 4.** Multivariable Model Performance **eTable 5.** Nomogram Scoring Tables
eReferences
