# Supplementary material for: Estimating Risk of Chronic Pain and Disability Following Musculoskeletal Trauma in the United Kingdom
Source: JAMA Netw Open. 2022 Aug 26;5(8):e2228870. doi: 10.1001/jamanetworkopen.2022.28870 (PMC9419019; doi:10.1001/jamanetworkopen.2022.28870)
Supplement: Supplement. — eFigure 1. Chronic Pain Grade Scores at 6 Months and 12 Months Posttrauma eFigure 2. Calibration Plot for 6-Month Multivariate Model eFigure 3. Calibration Plot for 12-Month Multivariate Model eTable 1. Summary of Data Collected Within the Major Trauma Center eTable 2. Participant Baseline Characteristics by Participant Retention eTable 3. Participant Baseline Characteristics by Outcome eTable 4. Multivariable Model Performance eTable 5. Nomogram Scoring Tables eReferences [file jamanetwopen-e2228870-s001.pdf]

Supplemental Online Content

Evans DW, Rushton A, Middlebrook N, et al. Estimating risk of chronic pain and disability following musculoskeletal trauma in the United Kingdom. *JAMA Netw Open*. 2022;5(8):e2228870. doi:10.1001/jamanetworkopen.2022.28870

eFigure 1. Chronic Pain Grade Scores at 6 Months and 12 Months Posttrauma
eFigure 2. Calibration Plot for 6-Month Multivariate Model
eFigure 3. Calibration Plot for 12-Month Multivariate Model
eTable 1. Summary of Data Collected Within the Major Trauma Center
eTable 2. Participant Baseline Characteristics by Participant Retention
eTable 3. Participant Baseline Characteristics by Outcome
eTable 4. Multivariable Model Performance
eTable 5. Nomogram Scoring Tables
eReferences

This supplemental material has been provided by the authors to give readers additional information about their work.

© 2022 Evans DW et al. *JAMA Network Open.*

eFigure 1. Chronic Pain Grade Scores at 6 Months and 12 Months Posttrauma

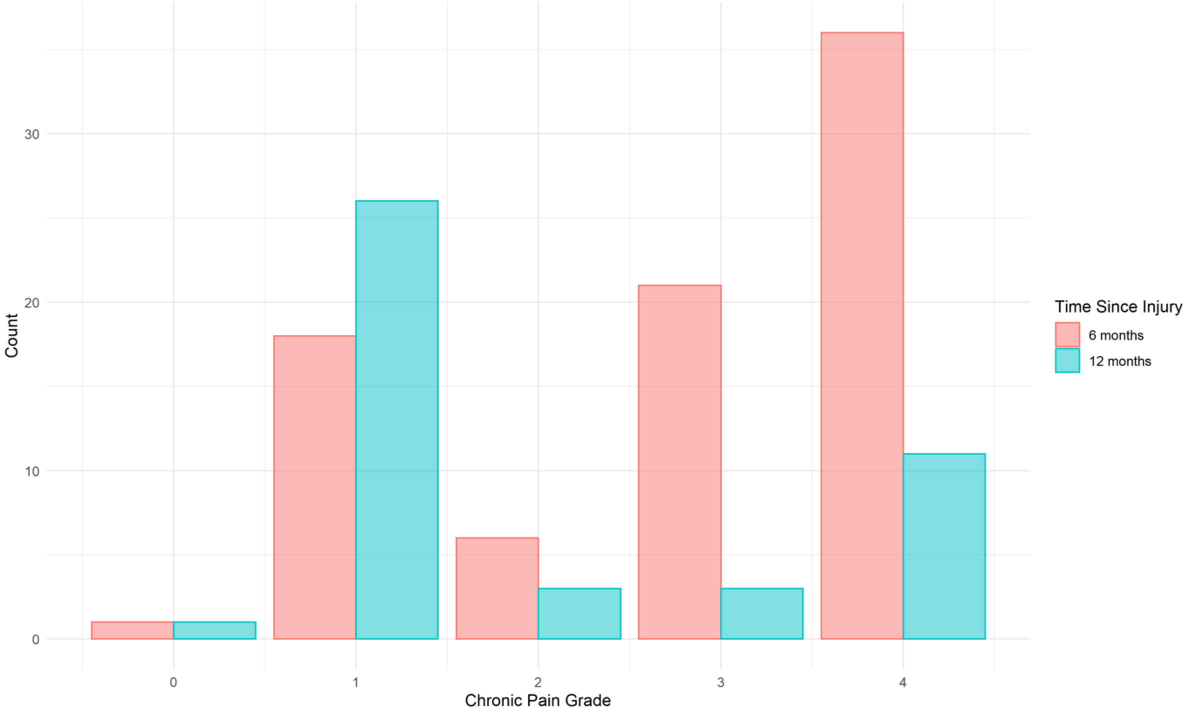

© 2022 Evans DW et al. *JAMA Network Open.*

eFigure 2. Calibration Plot for 6-Month Multivariate Model

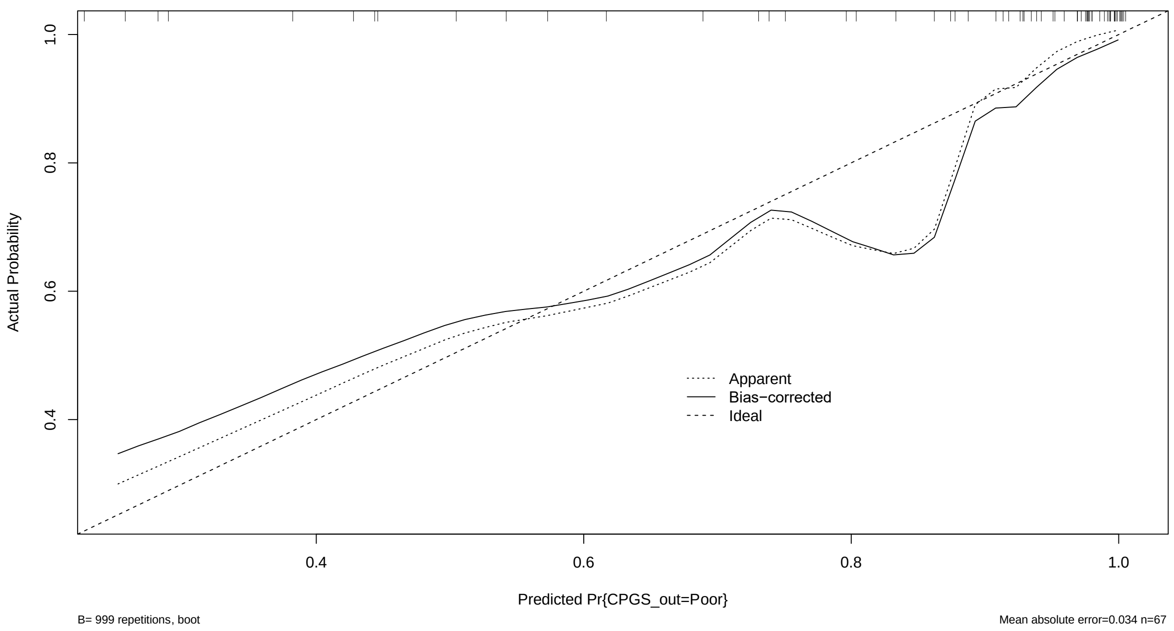

© 2022 Evans DW et al. *JAMA Network Open.*

eFigure 3. Calibration Plot for 12-month Multivariate Model

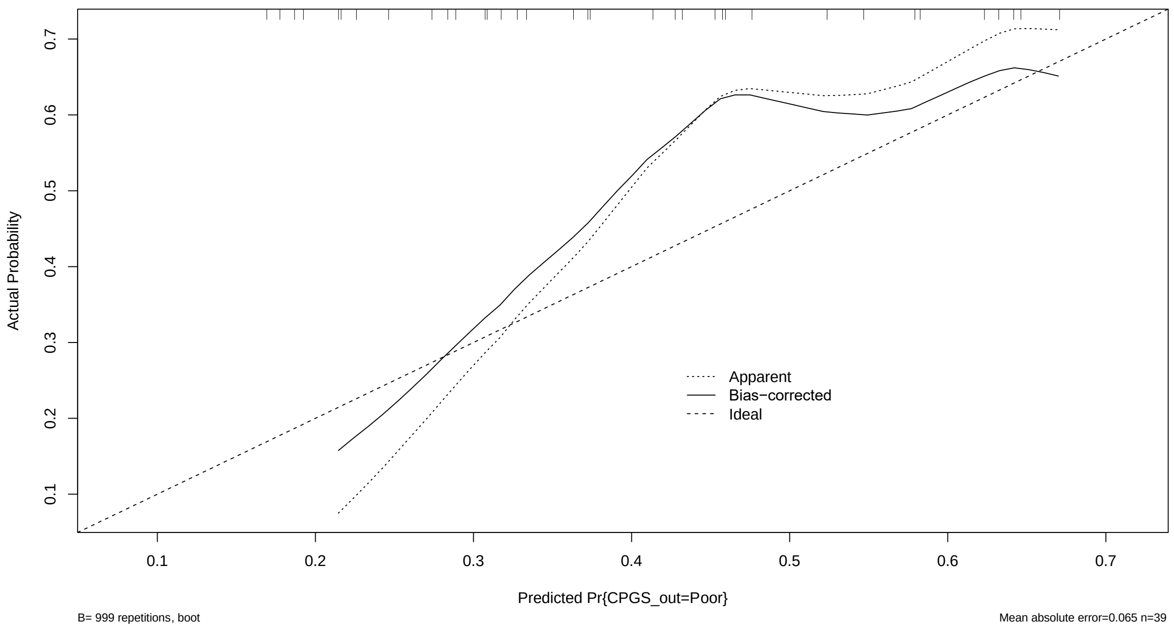

© 2022 Evans DW et al. *JAMA Network Open.*

eTable 1. Summary of Data Collected Within the Major Trauma Center

Domain	Measure	Baseline<br>*≤14 days post-trauma*	Follow-up<br>*3-, 6- and 12-months post-trauma*
**General patient characteristics**			
Age	Years	✓	
Gender assigned at birth	Female / male / not disclosed	✓	
Body Mass Index (BMI)	Calculated from height and weight measurements	✓	
Education	Highest educational level attained	✓	
Employment status	Full-time/ part-time / not working / retired / student<br>Employed / self-employed	✓	
Circumstance of trauma	Civilian / military	✓	
Previous history of musculoskeletal pain and injury	Patient history data from patient recollection and medical records	✓	
Comorbidity of other health problems	Patient history data from patient recollection and medical records	✓	
Premorbid physical health	Patient history data from patient recollection and medical records	✓	
Premorbid psychological health	Patient history data from medical records and patient recollection (including non-somatic items from the Subjective Health Complaints Inventory)[1]	✓	
Number of days in hospital	Intensive care / ward / total	✓	
**Injury characteristics**		✓	
Time of injury/incident	Hospital record data	✓	
Injury location	Adapted pain drawings, based on hospital record data	✓	
Tissues damaged	Based on imaging data and hospital records<br>Fractures (number, location)<br>Penetrating / non-penetrating injury / both	✓	
Surgery required	Location and post-injury timing of surgery, based on hospital record data	✓	
Assisted mechanical ventilation required	Yes (duration) / no	✓	
Severity of injury	Injury Severity Scale[2]	✓	
**Quality of life and physical functioning**			

© 2022 Evans DW et al. *JAMA Network Open.*

General health	36-item Short Form Health Survey, version 2 (SF-36v2)[3]	✓	✓
Health-related quality of life	EuroQol EQ-5D-5L[4]	✓	✓
Sleep quality<br>(past 24h, average since injury)	11-point (0-10) Numerical Rating Scales, relating to current pain, from 'best possible sleep'(0) to 'worst possible sleep' (10)[5]	✓	✓
Anxiety and depression	Hospital Anxiety and Depression Scales (HADS)[6]	✓	✓
Fear of movement or fear of injury or re-injury during movement	Tampa Scale of Kinesiophobia, 11-item (TSK-11)[7]	✓	✓
Confidence in ability to perform activities despite pain	Pain Self-Efficacy Questionnaire[8]	✓	✓
Post-traumatic stress (intrusion, avoidance and hyperarousal subscales)	Impact of Event Scale revised (IES-R)[9]	✓	✓
**Pain characteristics**			
Pain severity	Chronic Pain Grade Scale[10]		✓
Pain intensity<br>(current, average, worst and least)	11-point (0-10) Numerical Rating Scale, relating to current pain, from 'no pain' (0) to 'pain as bad as could be' (10) (from the Chronic Pain Grade Scale[10])	✓	✓
Self-reported features of neuropathic pain	painDETECT questionnaire[11]	✓	✓
Pain location	Pain drawing	✓	✓
Pain extent	Electronic pain drawing[12]	✓	
Pain medication intake<br>(type, dosage and timing)	Medication Quantification Scale,[13-15] based on hospital record data	✓	
**Quantitative sensory testing**		✓	
Heat pain threshold	Evaluation of pain threshold using a heat stimulus	✓	
Cold pain threshold	Evaluation of pain threshold using a cold stimulus	✓	
Pressure pain threshold	Evaluation of pain threshold using a pressure stimulus	✓	
**Biomarkers**			
C-reactive protein (CRP)	Serum levels of CRP, a broad indicator of inflammation<br>(via blood samples)	✓	

© 2022 Evans DW et al. *JAMA Network Open.*

eTable 2. Participant Baseline Characteristics by Participant Retention

	Responded at 6 months			Responded at 12 months		
**Characteristic**	**Yes**	**No**	***p*-value**	**Yes**	**No**	***p*-value**
Total, n (%)	67 (54.0)	57 (46.0)		39 (31.5)	85 (68.5)	
Demographics						
Age, mean (SD), y	50.11 (17.53)	47.42 (20.14)	0.428	52.89 (17.21)	47.03 (19.22)	0.106
BMI, mean (SD), kg/m^2	28.34 (6.70)	27.30 (5.84)	0.363	28.06 (7.11)	27.77 (5.95)	0.816
Female gender at birth, n (%)	27 (40.3)	17 (29.8)	0.305	16 (41.0)	28 (32.9)	0.502
Male gender at birth, n (%)	40 (59.7)	40 (70.2)		23 (59.0)	57 (67.1)	
White ethnicity, n (%)	57 (85.1)	47 (82.5)	0.881	35 (89.7)	69 (81.2)	0.346
Non-white ethnicity, n (%)	10 (14.9)	10 (17.5)		4 (10.3)	16 (18.8)	
Education age 16 or less, n (%)	26 (39.4)	22 (38.6)	0.720	14 (36.8)	34 (40.0)	0.800
Education age 17-19, n (%)	22 (33.3)	18 (31.6)		15 (39.5)	25 (29.4)	
Education age 20 or over, n (%)	15 (22.7)	15 (26.3)		8 (21.1)	22 (25.9)	
Still in full-time education, n (%)	3 (4.5)	1 (1.8)		1 (2.6)	3 (3.5)	
Preferred not to disclose education age, n (%)	0 (0.0)	1 (1.8)		0 (0.0)	1 (1.2)	
Working, n (%)	45 (67.2)	33 (57.9)	0.217	25 (64.1)	53 (62.4)	0.627
Not working, n (%)	22 (32.8)	22 (38.6)		14 (35.9)	30 (35.3)	
Preferred not to disclose work status, n (%)	0 (0.0)	2 (3.5)		0 (0.0)	2 (2.4)	
Smoker, n (%)	11 (16.4)	10 (17.5)	0.634	5 (12.8)	16 (18.8)	0.710
Non-smoker, n (%)	51 (76.1)	40 (70.2)		30 (76.9)	61 (71.8)	
Ex-smoker, n (%)	5 (7.5)	7 (12.3)		4 (10.3)	8 (9.4)	
Hospital data						
Hospital stay, mean (SD), d	18.33 (14.52)	16.65 (14.41)	0.521	19.03 (17.05)	16.88 (13.12)	0.445
ISS Total, mean (SD)	9.90 (7.36)	10.66 (8.60)	0.663	8.65 (5.83)	11.07 (8.69)	0.168
ISS Mild, n (%)	22 (42.3)	14 (40.0)	0.919	15 (48.4)	21 (37.5)	0.568
ISS Moderate, n (%)	20 (38.5)	13 (37.1)		11 (35.5)	22 (39.3)	
ISS Major, n (%)	10 (19.2)	8 (22.9)		5 (16.1)	13 (23.2)	
Number of fractures, mean (SD)	1.75 (1.20)	2.06 (1.29)	0.180	1.59 (1.07)	2.03 (1.30)	0.075
Penetrating injury, n (%)	49 (73.1)	38 (66.7)	0.557	30 (76.9)	57 (67.1)	0.366
Non-penetrating injury, n (%)	18 (26.9)	19 (33.3)		9 (23.1)	28 (32.9)	
General health measures						
EQ-5D-5L Index, mean (SD)	0.02 (0.32)	0.06 (0.31)	0.498	0.04 (0.31)	0.04 (0.32)	0.968
EQ-5D VAS, mean (SD)	43.76 (24.86)	47.39 (23.58)	0.409	48.08 (25.17)	44.21 (23.87)	0.412
SF36v2 Mental, mean (SD)	31.20 (8.50)	30.72 (6.65)	0.732	35.22 (14.25)	36.49 (13.02)	0.627
SF36v2 Physical, mean (SD)	35.83 (13.81)	36.38 (12.98)	0.820	31.70 (8.20)	30.64 (7.45)	0.479
Psychological measures						
HADS Anxiety, mean (SD)	8.22 (5.03)	7.60 (4.81)	0.497	8.13 (5.59)	7.84 (4.59)	0.765
HADS Depression, mean (SD)	7.75 (4.21)	7.36 (4.46)	0.620	7.62 (4.66)	7.55 (4.17)	0.936
TSK-11 Total, mean (SD)	28.27 (7.50)	25.85 (6.75)	0.073	27.71 (7.98)	26.93 (6.93)	0.592
PSEQ Total, mean (SD)	16.89 (14.72)	18.54 (15.44)	0.554	16.57 (15.56)	18.12 (14.82)	0.603
IES-R Total, mean (SD)	30.04 (21.59)	28.70 (19.97)	0.737	28.87 (21.00)	29.82 (20.93)	0.819

© 2022 Evans DW et al. *JAMA Network Open.*

	Responded at 6 months			Responded at 12 months		
**Characteristic**	**Yes**	**No**	***p*-value**	**Yes**	**No**	***p*-value**
IES-R Avoidance, mean (SD)	1.27 (0.98)	1.24 (0.88)	0.862	1.17 (0.97)	1.30 (0.92)	0.458
IES-R Hyperarousal, mean (SD)	1.32 (1.10)	1.26 (1.02)	0.772	1.29 (1.13)	1.29 (1.03)	1.000
IES-R Intrusion, mean (SD)	1.50 (1.09)	1.35 (0.98)	0.444	1.47 (1.04)	1.41 (1.05)	0.764
Pain measures						
Pain intensity (average), mean (SD)	4.67 (2.02)	4.87 (2.22)	0.688	4.65 (2.17)	4.80 (2.05)	0.740
Pain intensity (current), mean (SD)	3.62 (2.55)	3.75 (2.27)	0.807	3.48 (2.53)	3.77 (2.40)	0.602
Pain intensity (worst), mean (SD)	8.33 (2.08)	8.47 (2.18)	0.767	8.16 (2.42)	8.51 (1.92)	0.469
Pain intensity (least), mean (SD)	2.27 (2.36)	2.50 (2.50)	0.672	2.35 (2.46)	2.36 (2.39)	0.995
Pain extent, mean (SD), % pixels	8.07 (7.26)	7.06 (4.96)	0.511	5.69 (5.17)	9.00 (7.02)	0.026
Pain region count, mean (SD), number	5.45 (4.79)	5.28 (4.53)	0.843	4.79 (4.49)	5.64 (4.73)	0.353
PainDETECT Total, mean (SD)	13.59 (7.31)	13.87 (9.26)	0.884	13.54 (7.08)	13.79 (8.70)	0.903
Other measures						
Sleep quality last 24 hrs, mean (SD)	4.06 (2.50)	3.95 (2.66)	0.809	4.08 (2.28)	3.98 (2.70)	0.840
Sleep quality since injury, mean (SD)	3.69 (2.42)	3.30 (2.57)	0.388	3.64 (2.40)	3.45 (2.54)	0.689
Local heat pain threshold, mean (SD), °C	45.75 (3.56)	46.54 (3.30)	0.320	46.59 (3.02)	45.75 (3.68)	0.295
Remote heat pain threshold, mean (SD), °C	46.08 (3.66)	46.06 (3.60)	0.976	47.03 (3.21)	45.51 (3.75)	0.068
Local cold pain threshold, mean (SD), °C	5.81 (11.37)	2.95 (10.67)	0.267	6.21 (11.28)	3.89 (11.08)	0.369
Remote cold pain threshold, mean (SD), °C	6.64 (11.12)	3.25 (11.29)	0.191	4.78 (10.21)	5.74 (11.88)	0.714
Local pressure pain threshold, mean (SD), kPa	425.42 (198.56)	465.68 (225.04)	0.406	402.02 (161.10)	463.62 (230.66)	0.202
Remote pressure pain threshold, mean (SD), kPa	489.27 (236.39)	471.42 (243.15)	0.746	494.87 (229.24)	475.09 (244.55)	0.718
C-reactive protein, mean (SD), mg/L	48.43 (52.84)	56.17 (72.82)	0.551	53.15 (59.75)	50.95 (63.49)	0.870

© 2022 Evans DW et al. *JAMA Network Open.*

eTable 3. Participant Baseline Characteristics by Outcome

	6-months outcome			12-months outcome		
**Characteristic**	**Good**	**Poor**	**Difference (95% CI); p-value**	**Good**	**Poor**	**Difference (95% CI); p-value**
Total, n (%)	19 (23.2%)	63 (76.8%)		27 (61.4%)	17 (38.6%)	
Demographics						
Age, mean (SD), y	53.56 (17.61)	50.11 (17.80)	-3.44 (-12.55, 5.67)[1]; 0.459	53.29 (18.16)	57.12 (16.15)	3.83 (-6.74, 14.4)[1]; 0.477
BMI, mean (SD), kg/m^2	27.77 (2.72)	28.29 (7.01)	0.52 (-2.71, 3.75)[1]; 0.753	25.93 (3.30)	30.91 (9.42)	4.98 (1.12, 8.84)[1]; 0.012
Female gender at birth, n (%)	7 (36.8)	27 (42.9)	1	10 (37.0)	8 (47.1)	1
Male gender at birth, n (%)	12 (63.2)	36 (57.1)	-0.78 (0.26, 2.2)[2]; 0.639	17 (63.0)	9 (52.9)	0.66 (0.19, 2.28)[2]; 0.511
White ethnicity, n (%)	16 (84.2)	53 (84.1)	1	24 (88.9)	16 (94.1)	1
Non-white ethnicity, n (%)	3 (15.8)	10 (15.9)	1.01 (0.27, 4.89)[2]; 0.993	3 (11.1)	1 (5.9)	0.5 (0.02, 4.31)[2]; 0.546
Education age 16 or less, n (%)	8 (44.4)	26 (41.3)	1	8 (30.8)	9 (52.9)	1
Education age 17-19, n (%)	5 (27.8)	20 (31.7)	1.05 (0.40, 2.79)[3]; 0.922	11 (42.3)	4 (23.5)	0.5 (0.16, 1.61)[3]; 0.247
Education age 20 or over, n (%)	4 (22.2)	14 (22.2)		6 (23.1)	4 (23.5)	
Still in full-time education, n (%)	0 (0.0)	3 (4.8)		1 (3.8)	0 (0.0)	
Preferred not to disclose education age, n (%)	1 (5.6)	0 (0.0)		0 (0.0)	0 (0.0)	
Working, n (%)	13 (68.4)	40 (63.5)	1	16 (59.3)	11 (64.7)	1
Not working, n (%)	5 (26.3)	22 (34.9)	1.43 (-0.8, 1.51)[4]; 0.544	11 (40.7)	6 (35.3)	0.79 (0.22, 2.76)[2]; 0.717
Preferred not to disclose work status, n (%)	1 (5.3)	1 (1.6)	0.33 (-3.97, 1.72)[4]; 0.438	0 (0.0)	0 (0.0)	NA
Smoker, n (%)	3 (15.8)	11 (17.5)	1	4 (14.8)	2 (11.8)	1
Non-smoker, n (%)	15 (78.9)	45 (71.4)	0.82 (-1.61, 1.2)[4]; 0.779	20 (74.1)	13 (76.5)	1.3 (0.21, 8.15)[4]; 0.779
Ex-smoker, n (%)	1 (5.3)	7 (11.1)	1.91 (-1.81, 3.1)[4]; 0.606	3 (11.1)	2 (11.8)	1.33 (0.11, 15.7)[4]; 0.819
Hospital data						
Hospital stay, mean (SD), d	11.42 (5.86)	19.65 (15.89)	8.23 (0.91, 15.55)[1]; 0.027	13.89 (10.80)	25.65 (21.18)	11.76 (2.3, 21.22)[1]; 0.015
ISS Total, mean (SD)	7.88 (8.07)	10.95 (7.64)	3.08 (-1.35, 7.51)[1]; 0.173	8.78 (8.26)	10.00 (6.57)	1.22 (-3.95, 6.39)[1]; 0.643
ISS Mild, n (%)	10 (62.5)	15 (34.1)	3.44 (1.09, 10.84)[3]; 0.035	10 (55.6)	6 (40.0)	1.67 (0.46, 6.13)[3]; 0.437
ISS Moderate, n (%)	5 (31.2)	18 (40.9)		5 (27.8)	6 (40.0)	
ISS Major, n (%)	1 (6.2)	11 (25.0)		3 (16.7)	3 (20.0)	
Number of fractures, mean (SD)	1.19 (0.54)	1.98 (1.31)	0.8 (0.14, 1.45)[1]; 0.018	1.35 (0.89)	1.94 (1.20)	0.6 (-0.03, 1.22)[1]; 0.062

© 2022 Evans DW et al. *JAMA Network Open.*

	6-months outcome			12-months outcome		
**Characteristic**	**Good**	**Poor**	**Difference (95% CI); p-value**	**Good**	**Poor**	**Difference (95% CI); p-value**
Penetrating injury, n (%)	4 (21.1)	22 (34.9)	2.01 (0.64, 7.72)[2]; 0.242	7 (25.9)	3 (17.6)	0.61 (0.12, 2.63)[2]; 0.518
Non-penetrating injury, n (%)	15 (78.9)	41 (65.1)	1	20 (74.1)	14 (82.4)	1
General health measures						
EQ-5D-5L Index, mean (SD)	0.30 (0.25)	-0.04 (0.31)	-0.34 (-0.49, -0.18)[1]; <0.001	0.14 (0.32)	-0.06 (0.30)	-0.19 (-0.39, 0.00)[1]; 0.048
EQ VAS, mean (SD)	58.95 (21.19)	39.87 (22.38)	-19.07 (-30.42, -7.73)[1]; 0.001	51.30 (22.47)	43.82 (26.84)	-7.47 (-22.18, 7.23)[1]; 0.319
SF-36v2 Mental, mean (SD)	43.36 (12.95)	33.93 (12.76)	-4.48 (-8.59, -0.37)[1]; 0.032	36.03 (12.85)	33.82 (14.59)	-0.36 (-5.11, 4.38)[1]; 0.880
SF-36v2 Physical, mean (SD)	34.51 (9.55)	30.03 (7.44)	-9.42 (-16.02, -2.83)[1]; 0.005	31.70 (8.03)	31.33 (7.45)	-2.21 (-10.42, 6)[1]; 0.598
Psychological measures						
HADS Anxiety, mean (SD)	5.56 (3.91)	8.77 (4.89)	3.21 (0.75, 5.68)[1]; 0.011	7.70 (5.52)	8.29 (5.13)	0.59 (-2.67, 3.85)[1]; 0.723
HADS Depression, mean (SD)	5.42 (3.29)	8.22 (4.20)	2.8 (0.74, 4.86)[1]; 0.008	7.26 (4.36)	8.12 (4.88)	0.86 (-1.91, 3.63)[1]; 0.544
TSK-11 Total, mean (SD)	23.06 (7.67)	28.23 (7.32)	5.17 (1.19, 9.16)[1]; 0.011	25.96 (7.90)	27.60 (9.23)	1.64 (-3.79, 7.08)[1]; 0.554
PSEQ Total, mean (SD)	28.24 (13.38)	13.62 (13.72)	-14.61 (-21.95, -7.27)[1]; <0.001	22.92 (16.49)	9.56 (13.58)	-13.36 (-23.05, -3.67)[1]; 0.007
IES-R Total, mean (SD)	13.44 (11.09)	34.38 (21.16)	20.93 (10.72, 31.15)[1]; <0.001	22.44 (20.56)	35.12 (19.31)	12.68 (0.31, 25.04)[1]; 0.044
IES-R Avoidance, mean (SD)	0.55 (0.51)	1.45 (0.94)	0.9 (0.45, 1.36)[1]; <0.001	0.90 (0.90)	1.46 (0.92)	0.56 (0, 1.12)[1]; 0.049
IES-R Hyperarousal, mean (SD)	0.59 (0.69)	1.50 (1.08)	0.91 (0.39, 1.43)[1]; 0.001	0.99 (1.03)	1.66 (1.10)	0.66 (0.01, 1.31)[1]; 0.045
IES-R Intrusion, mean (SD)	0.82 (0.68)	1.65 (1.09)	0.83 (0.32, 1.35)[1]; 0.002	1.22 (1.04)	1.68 (0.96)	0.47 (-0.15, 1.08)[1]; 0.137
Pain measures						
Pain intensity (average), mean (SD)	3.50 (2.42)	5.07 (1.70)	1.57 (0.47, 2.66)[1]; 0.005	4.28 (1.81)	5.20 (2.46)	0.92 (-0.53, 2.38)[1]; 0.215
Pain intensity (current), mean (SD)	2.88 (2.45)	3.77 (2.56)	0.9 (-0.55, 2.35)[1]; 0.224	3.06 (2.13)	4.13 (2.77)	1.08 (-0.59, 2.75)[1]; 0.207
Pain intensity (worst), mean (SD)	6.56 (2.97)	8.95 (1.10)	2.39 (1.37, 3.41)[1]; <0.001	7.67 (2.40)	8.60 (2.56)	0.93 (-0.76, 2.63)[1]; 0.280
Pain intensity (least), mean (SD)	1.94 (2.49)	2.27 (2.18)	0.34 (-0.96, 1.63)[1]; 0.612	2.17 (2.31)	2.60 (2.59)	0.43 (-1.24, 2.1)[1]; 0.611
Pain extent, mean (SD), % pixels	2.73 (2.58)	9.56 (7.29)	6.82 (2.91, 10.74)[1]; 0.001	4.77 (4.95)	6.80 (5.19)	2.03 (-1.48, 5.55)[1]; 0.257

© 2022 Evans DW et al. *JAMA Network Open.*

	6-months outcome			12-months outcome		
**Characteristic**	**Good**	**Poor**	**Difference (95% CI); p-value**	**Good**	**Poor**	**Difference (95% CI); p-value**
Pain region count, mean (SD), number	2.53 (2.27)	6.17 (4.80)	3.65 (1.41, 5.88)[1]; 0.001	4.00 (3.76)	5.35 (5.28)	1.35 (-1.32, 4.02)[1]; 0.321
PainDETECT Total, mean (SD)	8.38 (5.45)	14.24 (7.54)	5.85 (1.35, 10.35)[1]; 0.011	10.69 (6.80)	17.89 (5.04)	7.2 (2.1, 12.3)[1]; 0.006
Other measures						
Sleep quality last 24 hrs, mean (SD)	4.05 (2.32)	3.90 (2.58)	-0.15 (-1.44, 1.15)[1]; 0.823	4.26 (2.31)	3.82 (2.92)	-0.44 (-1.99, 1.12)[1]; 0.583
Sleep quality since injury, mean (SD)	4.00 (1.97)	3.37 (2.57)	-0.63 (-1.89, 0.62)[1]; 0.321	3.81 (2.65)	3.53 (2.62)	-0.29 (-1.89, 1.32)[1]; 0.727
Local heat pain threshold, mean (SD), °C	46.21 (3.47)	45.70 (3.58)	-0.5 (-2.59, 1.58)[1]; 0.636	46.93 (2.84)	46.24 (3.11)	-0.68 (-2.75, 1.38)[1]; 0.517
Remote heat pain threshold, mean (SD), °C	46.03 (3.41)	45.96 (3.90)	-0.07 (-2.3, 2.16)[1]; 0.952	47.20 (2.63)	46.16 (4.03)	-1.04 (-3.35, 1.28)[1]; 0.38
Local cold pain threshold, mean (SD), °C	4.68 (10.12)	5.84 (11.97)	1.16 (-5.64, 7.95)[1]; 0.739	4.21 (10.85)	9.01 (10.79)	4.81 (-2.75, 12.37)[1]; 0.212
Remote cold pain threshold, mean (SD), °C	9.55 (10.93)	5.35 (11.53)	-4.2 (-10.91, 2.51)[1]; 0.220	5.24 (10.53)	5.19 (10.15)	-0.05 (-7.29, 7.19)[1]; 0.989
Local pressure pain threshold, mean (SD), kPa	427 (208)	444 (225)	17 (-117, 151)[1]; 0.803	376 (166)	404 (167)	28 (-87, 144)[1]; 0.631
Remote pressure pain threshold, mean (SD), kPa	515 (259)	485 (250)	-30 (-179, 118)[1]; 0.690	475 (215)	482 (259)	7 (-155, 168)[1]; 0.933
C-reactive protein, mean (SD), mg/L	38 (62)	54 (62)	16 (-20, 52)[1]; 0.382	63 (74)	53 (59)	-10 (-56, 36)[1]; 0.673

BMI = Body Mass Index; ISS = Injury Severity Scale; EQ-5D-5L = 5-level EuroQol 5-dimension; EQ VAS = EuroQol Visual Analogue Scale; SF-36v2 = 36-item Short Form Health Survey, version 2; HADS = Hospital Anxiety and Depression Scale; TSK-11 = Tampa Scale of Kinesiophobia; IES-R = Impact of Event Scale – Revised; PSEQ = Pain Self-Efficacy Questionnaire

[1]Output is the difference in means obtained from a linear regression model. Values <0 indicate lower average values in the Poor CPGS score group

[2]Output is the odds ratio obtained from a logistic regression model. Values of odds ratio<1 indicate lower event rates in the Poor CPGS score group

[3]Output is the odds ratio obtained from an ordinal logistic regression model. Values of odds ratio<1 indicate lower odds of moving to a 'higher' category in the Poor CPGS score group

[4]Output is the odds ratio obtained from a multinomial regression model. Values of odds ratio<1 indicate lower event rates in the Poor CPGS score group

© 2022 Evans DW et al. *JAMA Network Open.*

eTable 4. Multivariable Model Performance

		Observed at 6-months			Observed at 12-months		
		**Good outcome**	**Poor outcome**	**Total**	**Good outcome**	**Poor outcome**	**Total**
**Predicted**	**Good outcome**	7	4	11	18	8	26
	**Poor outcome**	6	50	56	4	9	13
	**Total**	13	54	67	22	17	39

© 2022 Evans DW et al. *JAMA Network Open.*

eTable 5. Nomogram Scoring Tables

Pain Intensity Average	Points
0	0
1	10
2	20
3	30
4	40
5	50
6	60
7	70
8	80
9	90
10	100
**Pain Extent at Baseline**	**Points**
0	0
5	14
10	27
15	41
20	54
25	68
30	82
35	95
**Number of Fractures**	**Points**
1	0
1.5	12
2	24
2.5	36
3	48
3.5	61
4	73
4.5	85
5	97
**IES-R Total**	**Points**
0	0
10	9
20	18
30	27
40	36
50	45
60	54
70	63

© 2022 Evans DW et al. *JAMA Network Open.*

80	73
**Total Points**	**Pr(Poor CPGS Score)**
1	0.05
34	0.20
54	0.40
71	0.60
92	0.80
125	0.95

© 2022 Evans DW et al. *JAMA Network Open.*

eReferences

1. Eriksen HR, Ihlebaek C, Ursin H. A scoring system for subjective health complaints (SHC). *Scand J Public Health.* 1999;27(1):63-72.
2. Baker SP, O'Neill B, Haddon W, Jr., Long WB. The injury severity score: a method for describing patients with multiple injuries and evaluating emergency care. *J Trauma.* 1974;14(3):187-196.
3. Ware J. SF-36 Health Survey Updated. *Spine.* 2000;25(24):3130-3139.
4. Brooks R. EuroQol: the current state of play. *Health policy (Amsterdam, Netherlands).* 1996;37(1):53-72.
5. Cappelleri JC BA, McDermott AM, Sadosky AB, Petrie CD, Martin S. Psychometric properties of a single-item scale to assess sleep quality among individuals with fibromyalgia. *Health Qual Life Outcomes.* 2009;17(7):54.
6. Zigmond AS, Snaith RP. The hospital anxiety and depression scale. *Acta Psychiatr Scand.* 1983;67(6):361-370.
7. Woby SR, Roach NK, Urmston M, Watson PJ. Psychometric properties of the TSK-11: a shortened version of the Tampa Scale for Kinesiophobia. *Pain.* 2005;117(1-2):137-144.
8. Nicholas MK. The pain self-efficacy questionnaire: Taking pain into account. *Eur J Pain.* 2007;11(2):153-163.
9. Beck JG, Grant DM, Read JP, et al. The impact of event scale-revised: psychometric properties in a sample of motor vehicle accident survivors. *J Anxiety Disord.* 2008;22(2):187-198.
10. Von Korff M, Ormel J, Keefe FJ, Dworkin SF. Grading the severity of chronic pain. *Pain.* 1992;50(2):133-149.
11. Freynhagen R, Baron R, Gockel U, Tolle TR. painDETECT: a new screening questionnaire to identify neuropathic components in patients with back pain. *Curr Med Res Opin.* 2006;22(10):1911-1920.
12. Barbero M, Mores, F., Leoni, D., Gatti, R., Egloff, M., Falla, D. Test-retest reliability of pain extent and pain location using a novel method for pain drawing analysis. *European Journal of Pain* 2015;19:1129-1138.
13. Masters Steedman S, Middaugh SJ, Kee WG, Carson DS, Harden RN, Miller MC. Chronic-pain medications: equivalence levels and method of quantifying usage. *Clin J Pain.* 1992;8(3):204-214.
14. Harden RN, Weinland SR, Remble TA, et al. Medication Quantification Scale Version III: update in medication classes and revised detriment weights by survey of American Pain Society Physicians. *J Pain.* 2005;6(6):364-371.
15. Gallizzi M GC, Harden RN, Stanos S, Khan A. Medication Quantification Scale Version III: internal validation of detriment weights using a chronic pain population. . *Pain Pract* 2008;8(1):1-4.

© 2022 Evans DW et al. *JAMA Network Open.*
